# A new genus and species of narrow-range millipede (Diplopoda, Polydesmida, Dalodesmidae) from Tasmania, Australia

**DOI:** 10.3897/zookeys.966.56308

**Published:** 2020-09-09

**Authors:** Robert Mesibov, Juanita Rodriguez

**Affiliations:** 1 West Ulverstone, Tasmania 7315, Australia Unaffiliated West Ulverstone Australia; 2 CSIRO, Australian National Insect Collection, Canberra, ACT, 2601, Australia Australian National Insect Collection Canberra Australia

**Keywords:** Australia, Dalodesmidae, Diplopoda, Polydesmida, Tasmania

## Abstract

*Kebodesmuszonarius***gen. nov. et sp. nov.** is only known from a small area on the Great Western Tiers in northern Tasmania, Australia, and like species of *Paredrodesmus* Mesibov, 2003 has no detectable paranota on the diplosegments. The gonopod telopodite of the new species is divided into a large, lateral, cowl-like structure, a solenomere and a medial branch with three processes.

## Introduction

The millipede described below is locally abundant and lives in easily accessed forest close to a major road. Nevertheless, it was overlooked in repeated faunal sampling in the area by the senior author and others until its discovery in 2019 during a flora and fauna survey conducted by the Department of Primary Industries, Parks, Water and Environment, Tasmania. Relationships of the new species are currently being studied using molecular genetic methods (Rodriguez et al., in prep.), as neither the gonopod structure nor the non-sexual character states suggest an affinity with any previously described Tasmanian Dalodesmidae.

## Materials and methods

**Figure 1.**
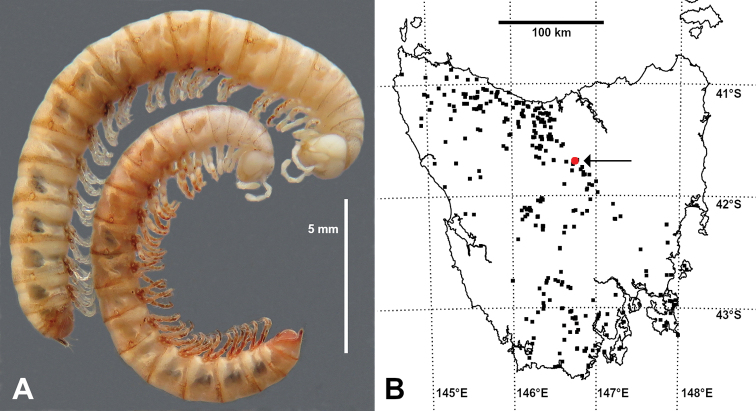
**A***Kebodesmuszonarius* gen. nov., sp. nov. female (top) and male (bottom) paratypes ex QVMAG QVM:2020:23:0003 after two days in 80% ethanol **B** mercator projection of Tasmania with *K.zonarius* gen. nov., sp. nov. localities (red circle marked with arrow) and *Paredrodesmus* localities with spatial uncertainty ±1 km or less (black squares). Localities for named *Paredrodesmus* species are from the Atlas of Living Australia (https://www.ala.org.au/) and for undetermined *Paredrodesmus* (females and juveniles) from the QVMAG collection database.

For analysis of defensive secretion, several specimens were gently picked up with soft forceps in the field and dropped into 1–2 ml of ca 100% methanol in a screw-capped glass vial. Other specimens were taken live to the laboratory and preserved in ca 95% ethanol or RNAlater (Ambion, Inc) for molecular analysis, or in ca 80% ethanol for examination, photography and museum storage. Specimens for genetic and chemical analysis are deposited in the Australian National Insect Collection, Canberra, and all others (in 80% ethanol) in the Queen Victoria Museum and Art Gallery, Launceston. Ring 7 of a male (ANIC 64:000360) was dissected, air-dried and dry-mounted on a conductive carbon tab attached to a stub for scanning electron microscopy. Scanning electron micrographs were obtained using a Hitachi TM3030Plus tabletop microscope at 5 KV with mixed imaging (backscatter + secondary electron). Gonopods of two paratype males were cleared in ca 80% lactic acid, temporarily mounted in a 1:1 glycerol:water mixture and imaged using an eyepiece video camera mounted on an Amscope binocular microscope. Focus-stacked images were assembled into composites with Zerene Stacker software version 1.04. Preliminary drawings were traced from printed copies of the composite images, then corrected by reference to the actual gonopods. Figures were composed using GIMP 2.10 and the map in Fig. 1B with QGIS 2.18.

Locations in the text are given in decimal degrees based on the WGS84 datum and were determined in the field with a handheld GPS unit. Elevations a.s.l. are from a topographic layer in LISTmap (https://maps.thelist.tas.gov.au/listmap/app/list/map). Specimen locality data are also provided in Suppl. material 1 in Darwin Core format.

Abbreviations: **ANIC**Australian National Insect Collection, Canberra, Australian Capital Territory, Australia; **QVMAG** Queen Victoria Museum and Art Gallery, Launceston, Tasmania, Australia.

## Results

### Order Polydesmida Pocock, 1887

#### Suborder Dalodesmidea Hoffman, 1980

##### Family Dalodesmidae Cook, 1896

###### Kebodesmus

Taxon classificationAnimaliaPolydesmidaDalodesmidae

Mesibov & Rodriguez gen. nov.

AAE7D3E4-66E9-57E7-9D74-C51DC9341165

http://zoobank.org/0B0CE131-A35A-4C03-85B4-FB2834CA7BDC

####### Type species.

*Kebodesmuszonarius* sp. nov., by present designation.

####### Other assigned species.

None.

####### Diagnosis.

Closely similar in general appearance to species of *Paredrodesmus* Mesibov, 2003, but distinguished from *Paredrodesmus* species in having H+20 body plan rather than H+19; normal pore formula rather than 5, 7–18; sphaerotrichomes on legs rather than no sphaerotrichomes; dorsal spinnerets within depression below epiproct tip rather than on epiproct rim; and a phenolic defensive secretion rather than no odour detectable from living specimens. Distinguished from all other Tasmanian Dalodesmidea (apart from *Paredrodesmus*) by the complete absence of paranota or traces of paranota on the diplosegments, and by the deep division of the gonopod telopodite.

####### Description.

As for the type species.

####### Name.

In honour of Kevin Bonham (Ke – bo), Tasmanian naturalist, collector and identifier, who emailed the senior author in May 2020 to say he had collected a millipede “whose gonopods I couldn’t even remotely match to anything”.

####### Remarks.

In gonopod structure *Kebodesmus* gen. nov. is unlike any of the undescribed Dalodesmidae so far examined in mainland Australian collections, and unlike any of the New Zealand Dalodesmidae described by Johns (1964, 1970). The gonopod in the new species is similar to that of *Abatodesmusvelosoi* Demange & Silva, 1971, a H+20 dalodesmid from the Cordillera de Nahuelbuta in southern Chile, but differs in having the solenomere base clearly separated from the other telopodite processes.

###### Kebodesmus zonarius

Taxon classificationAnimaliaPolydesmidaDalodesmidae

Mesibov & Rodriguez sp. nov.

2E795974-A037-5513-BB06-E013081A12C6

http://zoobank.org/348F8840-84F7-428D-8541-5D783B95F659

Figures 1A , 2

####### Holotype.

Male, Mountain Road (State Forest), Great Western Tiers, Tasmania, Australia, -41.6830, 146.7434 ± 25 m, 820 m a.s.l., 2 June 2020, R. Mesibov, QVMAG QVM:2020:23:0001.

####### Paratypes.

All from Great Western Tiers, Tasmania, Australia: 9 males, 5 females, details as for holotype, QVMAG QVM:2020:23:0002; 2 males, 2 females, Mountain Road (private property), -41.6855, 146.7515 ± 25 m, 770 m a.s.l., 29 May 2020, R. Mesibov, in 95% ethanol, ANIC 64:000351–64:000354; 9 males (2 dissected), 15 females (1 dissected), same details, QVMAG QVM:2020:23:0003; 1 male, 1 female, Mountain Road (private property), same details but -41.6865, 146.7509 ± 25 m, 780 m a.s.l., in 95% ethanol, ANIC 64:000355–64:000356; 1 male, Mountain Road, -41.6867, 146.7485 ± 25 m, 800 m a.s.l., 12 November 2019, K. Bonham, QVMAG QVM:2020:23:0004.

####### Other material.

3 females, Mountain Road (private property), -41.6865, 146.7509 ± 25 m, 780 m a.s.l., 29 May 2020, R. Mesibov, in methanol, ANIC 64:000357–64:000359; 5 males, 2 females, Mountain Road (State Forest), -41.6830, 146.7434 ± 25 m, 820 m a.s.l., 2 June 2020, R. Mesibov, in methanol, ANIC 64:000360–64:000366; 6 females, same details but in RNAlater, ANIC 64:000367–64:000372.

####### Description.

Male and female (Fig. 1A) both with head + 19 body rings + telson (H+20). Male/female approximate measurements: length 17/20 mm, midbody vertical diameter 1.4/1.9 mm. Freshly collected specimens pale yellow to pale reddish-brown, with reddish-brown speckling concentrated at rear of body rings and in distinct halo around ozopore; head pale and telson reddish-brown.

Male with clypeus and frons sparsely setose, vertex bare. Antennal sockets separated by ca 2× socket diameter. Antenna short, slender, just reaching anterior margin of ring 3 when manipulated backwards; relative length of antennomeres 6>(2,3)>(4,5), antennomere 6 widest. Collum in dorsal outline with anterior and posterior margins subparallel, corners smoothly rounded; a few long setae near anterior collum margin. Head slightly wider than ring 2; ring widths 2–17 almost uniform, slightly narrower on rings 2–4. Body cylindrical, waist only slightly impressed in lateral view. Prozonites and metazonites bare, unsculptured; limbus a narrow, uniform lamella. No trace of paranota on diplosegments; paranotum on rings 2, 3 reduced to thin, narrow ridge low on ring, longer on ring 2. Ozopore small, round, opening laterally at ca 2/3 ring height close to posterior metazonite margin; pore formula 5, 7, 9, 10, 12, 13, 15–19 (normal). Spiracles on diplosegments small, round; anterior spiracle on slightly produced process; posterior spiracle about midway between leg bases. Sternites slightly longer than wide, with deep transverse and longitudinal impressions, sparsely setose. Legs slender, about as long as maximum ring diameter at midbody; anterior legs with prefemur only very slightly swollen dorsally; relative podomere lengths (femur, tarsus)>prefemur>(postfemur, tibia) on midbody legs; tarsus straight. Sphaerotrichomes on tarsus, tibia and postfemur of anterior legs; sphaerotrichome numbers rapidly diminishing posteriorly with only a few sphaerotrichomes on tibia and tarsus of posterior legs; sphaerotrichome hemispherical with sharply pointed seta inclined distoventrally. No brush setae on any legs. Pre-anal ring with sparse, long setae; hypoproct trapezoidal; epiproct extending well past anal valves, tapering to truncate tip ca 1/6 maximum width of ring 19; spinnerets in square array in shallow cavity just ventral to epiproct tip.

Gonopore small, opening distomedially on leg 2 coxa. Bases of legs 6 and 7 well separated by shallowly concave sternite with sparse long setae. Aperture ovoid, wider than long, about 1/3 width of ring 7 prozonite, rim not produced. Gonocoxae subcylindrical, lightly joined basomedially; posteromedially with broad oblique depression bearing very short setae, cannula arising at distomedial end of depression, looping basally and inserted into deep, wide anterobasal groove on telopodite.

**Figure 2.**
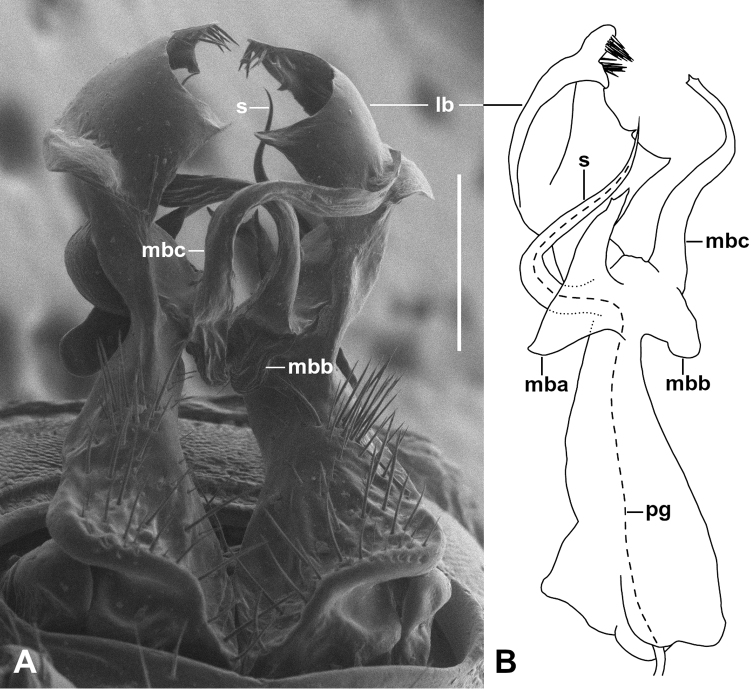
*Kebodesmuszonarius* gen. nov., sp. nov. **A** right posterolateral view of gonopods in situ; ANIC 64:000360 **B** medial view of left gonopod telopodite; paratype ex QVMAG QVM:2020:23:0003. **lb** = lateral branch, **mba** = process “a” of medial branch, **mbb** = process “b” of medial branch, **mbc** = process “c” of medial branch, **pg** = prostatic groove, **s** = solenomere. Scale bars: 0.25 mm (**A**, **B**).

Telopodite (Fig. 2) extending just past leg 6 bases when retracted, erect, divided at ca 1/2 telopodite height into a solenomere and complicated medial and lateral branches. Telopodite base below branching point subconical, posterolaterally depressed with field of long setae, at branching point on medial surface with rounded, tab-like extension.

Solenomere arising from anterior surface of telopodite, subcircular in cross-section, bending posteriorly at base, then slightly laterally, tapering gradually to sharply pointed tip at ca 7/8 of telopodite height.

Medial branch of telopodite divided into three processes. Medial processes from anterior to posterior: (a) large, thin, “fishtail”-shaped process bent anteriorly with rounded distal and basal margins, the distal tip of process approaching solenomere but bent away from it and terminating at ca 2/3 solenomere height; (b) short, thick, tab-like process directed posterobasally, terminating at same level as lower portion of process (a); (c) long, flattened process arising posteriorly, bending distomedially, then anterodistally, gradually tapering with minutely tricuspid apex distal to tip of solenomere and basal to tip of lateral branch of telopodite. Right and left (c) processes cross each other in gonopods in situ (Fig. 2A).

Lateral branch of telopodite expanded distally into lamellar, cowl-like structure, concave posteromedially, with two groups of apical marginal teeth; posterior margin of cowl produced as large triangular tooth approaching process (c) of medial branch of telopodite; anterior margin of cowl produced as large, rounded tab approaching solenomere; anterolateral (convex) surface of cowl with oblique, triangular tab.

Prostatic groove running from anterobasal groove along anteromedial surface of telopodite to base of solenomere, then along solenomere to tip.

Female resembling male but distinctly stouter (Fig. 1A). Genital aperture with posterior margin produced as rounded triangle medially; cyphopods not examined.

####### Name.

Latin *zonarius*, zonal, adjective. This species appears to be restricted to a narrow altitudinal zone on Tasmania’s Great Western Tiers.

####### Distribution and ecology.

So far known from four sites in wet eucalypt forest at ca 800 m a.s.l. on the Great Western Tiers in northern Tasmania, south of the town of Deloraine, with a linear range extent of less than 1 km (Fig. 1B). Adults and juveniles are found in patches of richly organic soil and humus in the forest, which is dominated by Eucalyptusdelegatensissubsp.tasmaniensis Boland. The new species co-occurs in humus with the native dalodesmids *Lissodesmusalisonae* Jeekel, 1984 and *L.perporosus* Jeekel, 1984, but was found in greater numbers than the other two species during searches in 2020.

####### Remarks.

*Kebodesmuszonarius* gen. nov., sp. nov. closely resembles a *Paredrodesmus* species in appearance and habits, and the new species occurs just on the eastern edge of the *Paredrodesmus* range (Fig. 1B). Although a juvenile *Paredrodesmus* was collected together with the new species at the 2019 site (QVMAG QVM:2020:23:0008), the senior author saw no *Paredrodesmus* individuals while searching for the new species in 2020. The interesting juxtaposition of ranges suggests that the two genera may exclude each other as competitors in the humus microhabitat.

Only a few of the adult *K.zonarius* gen. nov., sp. nov. collected in 2020 produced a strongly pungent defensive secretion in the field, even when disturbed, but the alcohol in which 2019 and 2020 specimens were first preserved had a phenolic smell.

## Supplementary Material

XML Treatment for Kebodesmus

XML Treatment for Kebodesmus zonarius

## Supplementary materials

Supplementary material 1 Specimen data for *Kebodesmuszonarius* gen. nov. et sp. nov. Data type occurrence Explanation note Data file Specimen_data_Kebodesmus_zonarius_2020.tsv for 9 specimen lots of *Kebodesmuszonarius* gen. nov. et sp. nov. in the Queen Victoria Museum and Art Gallery, Launceston, Tasmania, Australia and the Australian National Insect Collection, Canberra, Australian Capital Territory, Australia. The file is a tab-separated table in UTF-8 encoding with the following Darwin Core fields: institutionCode, catalogNumber, phylum, class, order, family, genus, specificEpithet, scientificName, typeStatus, organismRemarks, preparations, locality, locationRemarks, country, stateProvince, decimalLatitude, decimalLongitude, geodeticDatum, coordinateUncertaintyInMeters, georeferenceSources, georeferencedBy, minimumElevationInMeters, recordedBy, occurrenceRemarks and eventDate. https://binary.pensoft.net/file/450687This dataset is made available under the Open Database License (http://opendatacommons.org/licenses/odbl/1.0/). The Open Database License (ODbL) is a license agreement intended to allow users to freely share, modify, and use this Dataset while maintaining this same freedom for others, provided that the original source and author(s) are credited.Robert Mesibov, Juanita Rodriguez
